# Theory and Practice of Midwifery

**Published:** 1848

**Authors:** 


### ARTICLE III.

*Theory and Practice of Midwifery.* By FLEETWOOD CHURCHHILL, M.D., M. R. I. A.; Licentiate of the College of Physicians in Ireland; Physician to the Western Lying-in Hospital; Lecturer on Midwifery, &c., in the Richmond Hospital School of Medicine; Author of a "Treatise of the Diseases of Females," etc., etc., with Notes and additions, by ROBERT M. HUSTON, M.D., Professor of Materia Medica and General Therapeutics, and formerly of Obstetrics, &c., in Jefferson Medical College of Philadelphia, &c., &c. Third American edition with one hundred and twenty-eight illustrations, &c., &c. Philadelphia: Lea & Blanchard, 1848. (From the Publishers—for sale by Joseph Keene, Jr., Chicago.)

The established reputation of this work, renders it unnecessary for us to say any thing in its commendation.

In the present edition, the author has taken especial pains to adapt it to the wants of the profession in America. It is one of the best works we have on the subject of which it treats. [E.

